# Iron status in HIV-1 infection: implications in disease pathology

**DOI:** 10.1186/1472-6890-12-26

**Published:** 2012-12-17

**Authors:** S Olatunbosun Banjoko, Falilat A Oseni, Rachel A Togun, Olaniyi Onayemi, Beatrice O Emma-Okon, Julius B Fakunle

**Affiliations:** 1 Department of Chemical Pathology, College of Health Sciences, Obafemi Awolowo University, Ile-Ife, Nigeria; 2 Department of Haematology & Immunology, College of Health Sciences, Obafemi Awolowo University, Ile-Ife, Nigeria; 3 Department of Dermatology & Venereology, College of Health Sciences, Obafemi Awolowo University, Ile-Ife, Nigeria; 4 Department of Medical Biochemistry, College of Health Sciences, Obafemi Awolowo University, Ile-Ife, Nigeria; 5 Institute of Public Health, College of Health Sciences, Obafemi Awolowo University, Ile-Ife, Nigeria

**Keywords:** Iron metabolism, HIV-1 infection, Pathogenesis, Prognosis, Antioxidant, Free radicals

## Abstract

**Background:**

There had been conflicting reports with levels of markers of iron metabolism in HIV infection. This study was therefore aimed at investigating iron status and its possible mediation of severity of HIV- 1 infection and pathogenesis.

**Method:**

Eighty (80) anti-retroviral naive HIV-1 positive and 50 sero-negative controls were recruited for the study. Concentrations of serum total iron, transferrin, total iron binding capacity (TIBC), CD_4_^+^ T -lymphocytes, vitamin C, zinc, selenium and transferrin saturation were estimated.

**Results:**

The mean CD_4_^+^ T-lymphocyte cell counts, serum iron, TIBC, transferrin saturation for the tests and controls were 319 ± 22, 952 ± 57 cells/μl (P < 0.001), 35 ± 0.8, 11.8 ± 0.9 μmol/l (P < 0.001), 58.5 ± 2.2, 45.2 ± 2.4 μmol/l (P < 0.005) and 68.8 ± 3.3, 27.7 ± 2.2%, (P <0.001), respectively, while mean concentrations of vitamin C, zinc and selenium were 0.03 ± 0.01, 0.3 ± 0.04 (P < 0.001), 0.6 ± 0.05, 11.9 ± 0.26 μmol/l (P < 0.001) and 0.1 ± 0.01, 1.2 ± 0.12 μmol/l (P < 0.001) respectively. Furthermore, CD_4_^+^ T-lymphocyte cell count had a positive correlation with levels of vitamin C (r = 0.497, P < 0.001), zinc (r = 0.737, P < 0.001), selenium (r = 0.639, P < 0.001) and a negative correlation with serum iron levels (r = −0.572, P < 0.001).

**Conclusion:**

It could be inferred that derangement in iron metabolism, in addition to oxidative stress, might have contributed to the depletion of CD_4_^+^ T cell population in our subjects and this may result in poor prognosis of the disease.

## Background

The human immunodeficiency virus 1 (HIV – 1) infection is known to affect virtually all the organs of the body causing different metabolic derangements in addition to the depression of the immune system [1]. These metabolic derangements include oxidative stress due to persistent immune activation associated with uncontrolled HIV-1 replication leading to excessive reactive oxygen species (ROS) generation and metabolic acidosis [2-4]. Alterations in normal physiology as a result of immune response in HIV infections may also involve markers of iron metabolism [5,6]. In a complex mechanism yet to be unraveled, increased oxidative stress may result in the release of bound iron from their apoproteins therefore increasing the burden of plasma iron [7]. In addition, excessive free radical production associated with HIV – infection had been shown to participate in T – lymphocyte depletion by triggering apoptosis and cell death thereby weakening the immune system [8] and causing the impairment of the antiviral defenses mediated by T helper cells [9]. Furthermore, free radicals generation had also been shown to be one of the mediators of different metabolic alterations associated with progression of HIV infection and positively correlate with low concentrations of plasma zinc, selenium and vitamin E, an indication of possible antioxidant depletion [10-13].

Iron is an essential trace element present in biological systems in either ferrous (Fe^2+^) or ferric (Fe^3+^) state. Transferrin, a β1 glycoprotein synthesized in the liver, binds iron in the ferric form and transports it from the storage site for utilization through a receptor mediated pathway. The potential for the body iron concentration to be raised in HIV infection due to their accumulation in tissues such as liver, heart, pancreas and cells like macrophages is enormous and may result in free radical generation and accelerated catabolism of ascorbic acid [14-16]. Experimental evidence had shown that serum iron may be increased or decreased depending on the stage of the disease. Although iron stores may decline in early asymptomatic HIV infection probably because of impaired absorption [17], they may however increase with progression of the disease as iron accumulates in the macrophages and other cells [18]. Hepcidin, a hormone secreted by the hepatocytes is the key regulator of iron metabolism and acts by binding to the trans-membrane protein ferroportin responsible for the efflux transport of iron from the cells. Sequel to hepcidin binding to ferroportin, it is internalized into the cytoplasmic lysosomes and hydrolyzed thereby causing iron to accumulate in the cells with resultant hypoferraemia. Therefore, iron overload may occur when hepcidin is deficient due to various reasons including defect in gene expression of the protein or hepatic dysfunction [18,19]. Furthermore, potential availability of free iron due to abnormal physiology that may accompany iron overload can cause DNA double strand breaks and oncogene activation [20]. Investigating the putative links between iron status, disease progression and mortality is complicated and some confounding factors which include modification of iron status by the acute phase response (APR) with resultant redistribution of iron and paradoxical iron depletion in some body compartments and iron overload in others had been identified. These inconsistencies in impact of iron status on HIV infection, were therefore the underpinnings of this study [14,15,21-23].

## Methods

### Ethical clearance

Ethical clearance was obtained from Obafemi Awolowo University Teaching Hospital Complex Human Research Ethics Committee and informed consent was obtained from all the participants.

### Tests and controls

Eighty (80) patients including 48 females (60%) and 32 males (40%) aged between 20 and42 years attending the clinics of Obafemi Awowolo University Teaching Hospital Complex, Ile-Ife, Nigeria who were newly diagnosed as HIV-1 positive, at different stages of the infection and with no evidence of co-existing disease were recruited for the study. Most were asymptomatic and some were recruited from the routine screening at the ante-natal clinic. The subjects were recruited sequel to calculating the sample size, using the prevalence of 4.5% of the study area. Fifty (50) sero-negative individuals including 30 females (60%) and 20 males (40%) aged between 22 and 45 years were also recruited as controls. The exclusion criteria for all subjects included ingestion of supplements like vitamin C and ferrous preparations over a two-month period.

### Screening tests for HIV-1 and HIV-2

Two different HIV screening kits were used for the detection of sero-positivity using World Health Organization (WHO) criteria. A rapid ELISA was done on whole blood collected by finger prick using Determine^TM^ HIV1/2 kit (Abbot Laboratories, Japan, and STAT-PAK assay kit (Chembio Diagnostic Systems, USA). The principle of the two test methods is based on antigen-antibody reactions with colour indicators. Samples were regarded as positive with a color change and negative without it.

### Collection of sample

Ten milliliters of venous blood was collected from each participant into vacutainer tubes. Five milliliter of the blood was placed in both EDTA and plain sample bottles. Serum obtained from clotted blood was placed into the plain bottle after clot retraction and centrifugation at 25,000 rpm for five minutes using a bench centrifuge. The serum sample was thereafter stored frozen at –70°C for later analysis for iron, transferrin, total iron binding capacity (TIBC), zinc, selenium and vitamin C. The blood sample in the EDTA bottle was used immediately after collection for the determination of CD^+^_4_ T-lymphocyte count.

### Determination of CD^+^_4_ T-lymphocyte count

CD^+^_4_ T-lymphocyte count was done using Partec Cyflow counter and Partec CD^+^_4_ T-lymphocyte easy count kit. Twenty micro liters of whole blood was placed in a test tube, thereafter, 20 μl of CD4- MAB/PE (dye) was added and vortex -mixed gently. The mixture was then incubated for 15 min at room temperature. To this mixture, 80 μl of no-lyse buffer was added and mixed. The pre-stained mixture was aspirated into the Cyflow counter. The count result was displayed electronically.

### Determination of serum iron concentration

Serum iron was determined by the method of Garcic (1979). The method is based on the formation of ternary complex: chromazurol B (CAB) – cetyl-trimethyl ammonium bromide (CTMA) - iron complex which was read in a spectrophotometer at 623 nm. The concentration of iron in samples and controls were determined from the interpolation of the absorbance on the known concentration of standard, as follows:

$$\frac{\text{Absorbance}\;\mathrm{of}\;\text{Test}\;\mathrm{or}\;\text{Control}}{\text{Absorbance}\;\mathrm{of}\;\text{Standard}}\times 17.9\;\mu \frac{\text{mol}}{l}$$

### Estimation of total iron binding capacity (TIBC)

Serum TIBC was determined using the spectrophotometric method. Serum was treated with excess ferric ions to ensure complete saturation of transferrin. Unbound excess iron was later adsorbed on aluminum oxide and precipitated. The ferrous ions in the supernatant were thereafter estimated by the previously described method for serum iron estimation. The TIBC was calculated as serum iron x 3 μmol/l. One milliliter of ferric chloride was added to 0.5 ml of serum in a tube and incubated at room temperature for 5 min, 1.0 ml of aluminium oxide (Alox) was added, the mixture was covered and placed in a vortex mixer for 10 min. The tubes were removed and allowed to stand for 3 min. The clear supernatant was used for iron estimation as described earlier.

### Transferrin saturation

Transferrin saturation was determined using the formula:

$$\frac{\text{Serum}\;\text{Iron}\;\left(\mu \frac{\text{mol}}{l}\right)}{\text{TIBC}}\times \frac{100}{1}\%$$

### Estimation of serum ascorbic acid concentration

Ascorbic acid was estimated according to the spectrophotometric method of Henry et al. (1974). [24] Ascorbic acid in serum was oxidized to it’s de-hydro form by cupric sulfate. The de-hydro- ascorbic acid was then reacted with 2,4, di-nitro-phenylhydrazine in the presence of sulphuric acid to form a red colour whose intensity is proportional to the concentration of ascorbic acid and read at 520 nm. Ascorbic acid concentrations were determined by interpolation of the absorbance of the standard and its concentration.

$$\frac{\text{Absorbance}\;\mathrm{of}\;\text{Test}\;\mathrm{or}\;\text{Control}}{\text{Absorbance}\;\mathrm{of}\;\text{Standard}}\times \frac{2.\text{0mg}}{\mathrm{dl}}$$

### Determination of serum zinc and selenium levels

Serum zinc and selenium levels were determined using alpha - 4 flame atomic absorption spectrophotometer (AAS) according to standard methods. De-proteinisation was done by placing 1.0 ml of serum in the test tube and adding 3 ml of 2 M HCl. The clear supernatant was aspirated into the flame atomic absorption spectrophotometer (AAS) after adjusting the wavelength at 213.9 nm and 196.1 nm for zinc and selenium estimation respectively. The concentrations were displayed electronically and the results were expressed in μmol/l.

### Statistical analysis

Data collected were analyzed using descriptive and inferential statistics. Student’s T- test, Kruskaal Wallis and Pearson’s correlation were the statistical methods employed and Statistical Package for Social Sciences (SPSS) software was utilized for these methods.

### Study limitations

This was a retrospective rather than prospective study making it difficult to obtain a sequential information on iron status at different stages of the infection.

## Results

### Background characteristics

The test group had eighty (80) subjects including 48 females (60%) and 32 males (40%) aged between 20 and 42 years with mean ages and standard deviation of 30.35 ± 5.47 years. The control group consisted of fifty (50) sero-negative controls including 30 females (60%) and 20 males (40%) aged between 22 and 45 years with mean age and standard deviation of 29.96 ± 6.31 years, t=0.373, (P=0.71). HIV-1 infection cuts across the socio-economic strata in Nigeria, therefore socio-economic status was not considered a confounder in the study.

### Markers of iron metabolism and immune status

The CD^+^_4_ T lymphocytes counts in our test subjects were significantly lower than those of the controls [Table 1]. Serum iron, total iron binding capacity (TIBC) and transferrin saturation were significantly different between the test and the control, P < 0.001 [Table 1].

**Table 1:** **Mean concentrations, standard deviation and P values of markers of iron metabolism and CD**^**+**^_**4**_ **T cell count in tests and controls**

**Parameters**	**Tests n=80**	**Controls n=50**	**P value**
	**Mean ± SD**	**Mean ± SD**	
CD^+^_4_ T cells/μl	319.6 ± 22	951.5 ± 57	<0.0001*
Serum Iron (μmol/l)	35.3 ± 0.8	11.8 ± 0.9	<0.001*
TIBC (μmol/l)	58.5 ± 2.2	45.2 ± 2.4	<0.001*
Transferrin Saturation (%)	68.0 ± 3.3	27.7 ± 2.2	<0.0001*

Values were expressed as mean ± standard deviation (SD).

P values <0.05 is significant.

*P denotes statistically significant value.

Furthermore, in the test subjects, there were significantly lower values in the three antioxidants estimated viz vitamin C, P < 0.001, zinc, P < 0.001 and selenium P < 0.0001 [Table 2]. In addition, there was a positive correlation with CD4+ T cell count and negative correlation with serum iron levels [Table 3].

**Table 2:** Mean concentrations, standard deviation and P values of some serum antioxidants in tests and controls

**Antioxidants**	**Test n = 80**	**Control n = 50**	**P- value**
	**Mean ± SD**	**Mean ± SD**	
Vitamin C (μmol/l)	2.27 ± 0.57	17.03 ± 2.27	<0.001 *
Zinc (μmol/l)	0.6 ± 0.05	11.9 ± 0.26	<0.0001*
Selenium (μmol/l)	0.1 ± 0.02	1.2 ± 0.12	<0.0001*

Values were expressed as mean ± standard deviation (SD).

P values <0.05 is significant.

*P denotes statistically significant value.

**Table 3:** **Correlation between CD**_**4**_^**+ **^**T cells, serum iron and antioxidants in test subjects**

**Parameters**	**CD**_**4**_^**+**^**T cell count**	
	**Correlation**	**P -value**
Vitamin C (μmol/l)	r = 0.497	P <0.001*
Zinc (μmol/l)	r = 0.737	P <0.001*
Selenium (μmol/l)	r = 0.639	P < 0.001*
Serum Iron (μmol/l)	r = − 0.572	P < 0.001*

r values denotes degree of positive or negative correlation.

P value < 0.05 is statistically significant.

*P denotes significant positive or negative correlation.

## Discussion

The significant variations in serum iron, transferrin saturation and total iron binding capacity (TIBC) obtained in HIV-1 infected subjects were indications of derangement in iron metabolism. In addition, serum iron was found to have negative correlation with CD_4_^+^ T- lymphocytes [Table 3. These findings corroborated the results of some other workers who had previously observed that iron impaired the CD_4_^+^ T cell sub population *in vitro*[15,25]. High plasma iron and body iron stores have the potential of promoting free radical generation and oxidative stress via the popular Fenton/Haber –Weiss reaction [2,26-29]. The various metabolic derangements predispose HIV patients to metabolic acidosis [30] which promotes reduced binding of iron molecules to transferrin with resultant increase in serum free iron [7]. It is of importance to note that poorly liganded iron with resultant increased body free iron which readily reacts with free radicals, particularly hydroxyl radicals had been observed in many pathological processes and inflammatory diseases [41,42]. The possibility of occurrence of this scenario is therefore high in an event of metabolic acidosis of HIV infection.Furthermore, the fact that excessive free iron promotes oncogene activation and tumour proliferation underscores the possible involvement of derangement in iron metabolism in incidence of HIV associated cancers like Kaposi’s sarcoma and lymphomas [20].

Generation of free radicals has been implicated in the depletion of the antioxidant stores and progressive loss of CD_4_^+^ T helper cells, which is indicated by the significantly lower levels of vitamin C obtained in our test subjects. In this study, the fact that CD_4_^+^ T cell counts correlated positively with the antioxidant levels [Table 3, and supplementation with antioxidants such as zinc and selenium have been shown to improve the immune status in HIV-1 patients supports their usefulness as adjuvants to antiretroviral therapy [31-34]. However, the low antioxidant status observed in our study can also be attributed to generalized cachexia, nutrient malabsorption and diarrhea commonly found in HIV-1 infections.

Another dimension of consequences of increased plasma iron is the fact that iron- rich environment not only renders the patients more susceptible to microbial infections like *Mycobacterium tuberculosis, Candida albicans, Salmonella* and hepatitis viruses *B* and *C* but promotes their proliferation with a significant contribution to the morbidity and mortality that accompanies HIV-1 infection.

The burden of elevated plasma iron can be further compounded by alcohol ingestion, iron and multivitamins supplementation. Alcohol not only contains iron, but aids the absorption of iron from the intestines [7]. The synergistic effect of iron supplementation and alcohol consumption in HIV infection can therefore be better imagined. It is also pertinent to note that iron supplements and iron containing multivitamins are sold off the counters in pharmacies and their usage without prescription are common practices particularly in developing countries.

Other possible contributor to raised plasma iron is a condition termed African iron overload, with distinct features from the well characterized HLA linked haemochromatosis observed in Caucasians and iron overload unexplained by dietary, medicinal or excessive blood transfusion with clinical significance which had been observed in Africans and Americans of African descents[35-39]. It is therefore plausible to suggest an interaction between an unidentified gene and dietary iron content in addition to derangement in intracellular iron metabolism to be possible contributors to the elevated serum iron observed in the patients studied. Our results however confounded our expectation which the burden of helminthiasis and malnutrition common in Africa could have on serum iron levels. This underscores the clinical significance of this study.

Although the retrospective nature of this study was a limitation, there was no concurrence in the association of iron status with severity of the disease in other prospective and retrospective studies. For example, no correlation was observed between plasma iron levels and markers of severity of HIV disease in some Malawian sero-positive pregnant women [21]. Contrastly, in another cross sectional study of some sero-positive pregnant Zimbabwean women receiving iron supplementation, ferritin level was found to be an independent predictor of viral load [40]. Although evidences of elevated iron status in HIV infection were observed in some similar retrospective studies [5,6,40], this was however not the case in many others [21,23]. Furthermore, associations between iron accumulation and such adverse conditions like anaemia commonly unresponsive to iron supplementation, observed to increase the incidence of opportunistic infection and shorter survival periods led credence to the critical role of iron metabolism in the pathogenesis of HIV disease [6,11].

From all indications, the virus-host iron status interaction is yet to be fully understood. While some viruses selectively infect iron acquiring cells by binding to transferrin receptor-1 during cell entry, others alter the expression of proteins involved in iron homeostasis such as human haemochromatosis protein; (HFE) and hepcidin. Therefore iron overload associated with poor prognosis in HIV-1 infection could be partly caused by the viruses themselves [18]. Plausibly, understanding the regulation of hepcidin production and how iron metabolism and viral infection interact may in the near future stimulate the development of new methods and strategies in the treatment and management of the disease.

## Conclusion

It could be inferred that derangement in iron metabolism with resultant increase in plasma and total body iron concentrations might have contributed to the depletion of CD_4_^+^ T cell population and the antioxidant stores. It is therefore plausible to suggest that iron supplementation and consumption of alcohol should be discouraged in HIV infection, while antioxidant supplementation could be recommended as adjuvant to antiretroviral therapy. In addition, consideration should be given to periodic evaluation of total antioxidant status (TAS), acid–base balance and serum iron in the treatment and management of HIV-1 infection. This may likely improve the prognosis and immune reconstitution post anti-retroviral therapy. It is equally expedient to suggest the consumption of foods like brightly coloured fruits, vegetables, green teas and blue berries which are good sources of natural iron chelators capable of mitigating excessive release of free iron from their ligands by HIV infected individuals.

## Competing interests

The authors declare that they have no competing interests.

## Authors’ contributions

FAO: carried out the biochemical and haematological analyses and took part in the recruitment of patients. RAT: was responsible for the review of laboratory results, statistical analyses and the manuscript. SOB; conceived the idea, interpreted the data and was responsible for the write up of the manuscript. OO; was responsible for patients’ care, recruitment, clinical sorting and review of the manuscript, BOE; assisted in some biochemical analyses, JBF; was responsible for the overall supervision of the research. All authors read and approved the final manuscript.

## Pre-publication history

The pre-publication history for this paper can be accessed here:

http://www.biomedcentral.com/1472-6890/12/26/prepub
